# Exploratory Clinical Trial of Honghua Xiaoyao Pills for the Treatment of Liver Depression and Haemostasis‐Type Hyperplasia of Mammary Glands: A Single‐Centre, Single‐Arm Study

**DOI:** 10.1155/tbj/3499662

**Published:** 2026-06-24

**Authors:** Li Zheng, Wei Liu, Jia-Ying Qiu, Yang Zhao, Xiao-Feng Li, Zhi-Gang Zhou, Jiang-Tao Wang, Yu Cui, Xiao-Jun Zhang

**Affiliations:** ^1^ Xiyuan Hospital, Chinese Academy of Chinese Medical Sciences, Beijing, 100091, China, cacms.ac.cn; ^2^ Jiangxi Puzheng Pharmaceutical Co, Ltd., Ji’an, Jiangxi, 343199, China

**Keywords:** Honghua Xiaoyao pills, hyperplasia of mammary glands, liver depression and haemostasis

## Abstract

**Background:**

Hyperplasia of mammary glands (HMG) is a common benign disease of the female breast.

**Objective:**

This study aimed to observe the clinical efficacy and safety of Honghua Xiaoyao pills in the treatment of liver depression and haemostasis‐type HMG, as well as their impact on menstruation.

**Methods:**

Sixty patients diagnosed with liver depression and haemostasis‐type HMG in the breast disease department of our hospital were selected as the study participants. The treatment involved oral administration of Honghua Xiaoyao pills: four pills per dose, three times a day, for a duration of 3 months without interruption during menstruation. Hormones, Western painkillers and similar Chinese herbal medicines were prohibited during the trial. Changes in breast pain, breast masses, menstrual volume and safety indicators were observed.

**Results:**

Three patients were lost to follow‐up after 2 months of treatment, and four patients voluntarily withdrew from the study. The changes in breast pain scores, breast mass size scores and breast mass texture scores relative to baseline and their respective change rates showed statistically significant differences (*p* < 0.0001). Changes in lower abdominal distension, breast distension, menstrual blood clots, chest tightness/discomfort and emotional scores relative to baseline were also statistically significant (*p* < 0.0001).

**Conclusion:**

Honghua Xiaoyao pills are effective in reducing breast pain, shrinking breast masses and improving lower abdominal distension, breast distension, menstrual blood clots, chest tightness/discomfort and emotional symptoms in patients with liver depression and haemostasis‐type HMG.

**Trial Registration:** International Traditional Medicine Clinical Trial Registry: ITMCTR2024000165

## 1. Introduction

Hyperplasia of mamm glands (HMG) is a common benign disease of the female breast, characterised by abnormal growth and development of breast tissue but not classified as inflammation or tumour [1]. HMG typically manifests as increased breast tissue, the formation of nodules and possibly symptoms such as breast pain. Approximately 75% of women experience some degree of HMG, with approximately 20% of women being bothered by clinical symptoms. The mechanism of HMG is still not fully understood, although it is believed to be associated with various factors, such as hormonal imbalance and angiogenesis. Currently, the elevated levels of oestradiol or the imbalance between oestrogen and progesterone are considered to be closely related to HMG [2].

In Western medicine, the clinical treatment of HMG involves the use of various drugs, including thyroid preparations, prostate receptor antagonists and diuretics; however, their effectiveness is uncertain, leading to limited usage. Currently, hormone‐based medications, vitamins and iodine preparations remain the mainstay of treatment [3–5]. However, hormone receptors are widely distributed in multiple tissues, making patients prone to adverse reactions, such as menstrual disorders, irritability, nausea, liver damage and high recurrence rates after surgical treatment, causing significant trauma to patients. In Chinese medicine, HMG is classified as a ‘breast lump’ disease, with liver depression and emotional trauma playing a key role in its pathogenesis. Numerous studies have also explored the therapeutic effects of various Chinese herbal medicines in treating HMG [6, 7].

Honghua Xiaoyao pills have been on the market for many years and are mainly composed of Chinese angelica, white peony root, white atractylodes rhizome, *Poria cocos*, safflower, Chinese honeylocust spine, *Bupleurum marginatum*, mint and liquorice. They have the effects of soothing the liver, regulating qi and promoting blood circulation, making them suitable for treating liver depression and haemostasis‐type HMG [8]. However, there is limited research comprehensively evaluating its therapeutic effects and side effects in the treatment of patients with liver depression and haemostasis‐type HMG. In this study, Honghua Xiaoyao pills are used to treat 60 patients with liver depression and haemostasis‐type HMG to assess their effects and safety in relieving breast distension, masses, menstrual disorders and emotional abnormalities.

## 2. Materials and Methods

### 2.1. Study Population

A single‐centre, single‐arm, open‐label phase IV clinical study was conducted, including patients with liver depression and haemostasis‐type HMG who sought medical treatment at Xiyuan Hospital of China Academy of Chinese Medical Sciences between December 2021 and September 2022. The attending physician (who was also a member of the research team) was responsible for recruiting participants. Patients who met the inclusion and exclusion criteria of this study were informed in the local language by their attending physicians. The attending physician introduced the protocol of this study to the participants, including the research process, the risks of the study and the benefits of participating in it. Participants were informed that the examinations and medications designed for this study were free of charge. All participants signed informed consent forms before being included in the study.

The inclusion criteria were as follows: (1) meeting the diagnostic criteria for HMG in Western medicine, (2) meeting the syndrome differentiation criteria for liver depression and haemostasis in traditional Chinese medicine (TCM) and (3) women aged 18–45 years with regular menstrual cycles. The exclusion criteria were as follows: (1) pregnant or lactating women, peri‐menopausal women, intrauterine contraceptive device users and those with severe menstrual cycle disorders (e.g. hypogonadotropic hypogonadism, hyperprolactinemia, polycystic ovary syndrome or excessive menstrual bleeding causing anaemia), (2) abnormal uterine bleeding, organic lesions of the uterus and ovaries or decreased menstrual flow due to previous surgeries (e.g. intrauterine adhesions after uterine procedures or ovarian tumour resection), (3) patients with breast inflammation, malignant breast tumours or other breast diseases requiring surgical intervention, (4) breast masses > 1 cm in diameter on ultrasound or with a Breast Imaging‐Reporting and Data System score > 3, (5) use of contraceptive drugs or hormone medications within the past 3 months, (6) aged < 40 years with follicle‐stimulating hormone levels > 25 mIU/mL, (7) presence of thyroid dysfunction, (8) menstrual disorders caused by reproductive tract tumours, (9) severe primary diseases of the heart, liver, kidneys, haematopoietic system, gastrointestinal ulcers or mental illnesses, (10) allergies or hypersensitivity to the components of the study drug, (11) severe acute or chronic infections or secondary nephropathy, (12) patients planning pregnancy within the next 3 months, (13) participation in other clinical trials within the past 3 months or (14) suspected or confirmed history of alcohol or drug abuse or other conditions deemed by the investigator to reduce the likelihood of enrolment or complicate participation. The exit criteria were as follows: (1) voluntary withdrawal from research, (2) occurrence of adverse events where the researcher has determined that it is not suitable to continue participating in the study, (3) poor medication adherence, with medication usage < 80% during mid‐term evaluation, (4) use of other therapeutic medications during the study period or (5) other situations determined by researchers to be harmful to patient health or affect efficacy evaluation.

The study was conducted in accordance with the Clinical Research Guidelines for Chinese Herbal Medicine for the Treatment of Mammary Cystic Hyperplasia, the Diagnostic and Efficacy Standards for TCM Syndromes and Diseases in the People’s Republic of China and the content related to HMG in the third edition of Chinese Traditional Surgical Medicine. Specific indicators included the following: (1) breast pain, (2) breast masses and (3) the following auxiliary examinations: (i) breast colour Doppler ultrasound showing disordered or uneven enhancement of the breast tissue echoes, with visible ductal cystic dilatation, (ii) mammography showing increased density shadows with relatively uniform density in one or more quadrants of the breast and (iii) pathological examination showing varying degrees of proliferation and dilatation of mammary ducts and acini, as well as increased fibrous tissue in the stroma. The presence of items (1) and (2) was necessary, and the presence of any other three items was sufficient for the diagnosis of HMG.

Referring to the Guiding Principles for Clinical Research of Traditional Chinese Medicine New Drugs [9] and the Diagnostic, Dialectical and Therapeutic Evaluation Standards for Breast Hyperplasia discussed and revised by the Breast Disease Professional Committee of the Chinese Society of Traditional Chinese Medicine Surgery in 2007 [10], diagnostic criteria for liver depression and haemostasis‐type HMG were developed as follows: (1) main symptoms—(i) localised pain and swelling in the breast, with a fixed location, (ii) a palpable lump with unclear boundaries and a tough texture and (iii) tongue texture is purple and dark; (2) secondary symptoms—(i) breast pain is not significantly correlated with menstrual cycle or personal emotions, (ii) menstrual disorders, bloating during menstruation, poor menstrual flow, dark colour with blood clots and (iii) pulse string or astringency. Standard: diagnosis can be made with three main symptoms or two main symptoms plus two secondary symptoms.

Sixty patients were included according to the aforementioned inclusion and exclusion criteria. Among them, three patients were lost to follow‐up after 2 months of treatment (1 participant considered the efficacy to be poor and withdrew from the study to receive other treatments, and 2 participants believed that the pain had disappeared and there were no lumps when touching their breasts; they believed that they had recovered and did not need further treatment), and 4 patients were excluded due to personal reasons without undergoing medication observation (they did not take the medication as required by the plan, and the medication usage was < 80%). Among the remaining 53 patients, 51 were of Han ethnicity and 2 were of ethnic minorities. The average age was 29.41 ± 5.84 years, and the average body mass index was 21.3 ± 2.61 kg/m^2^. Forty‐two patients had no history of childbirth, and 11 patients had a history of childbirth and lactation. The average duration of the disease was 3.16 ± 3.5 years.

### 2.2. Data Collection

Patients’ demographics, medical history, present illness history, medication history, comorbidities, vital signs, menstrual history and completed symptom scoring forms were collected during the study. Blood routine, urine routine, urine pregnancy test, liver and kidney function, coagulation profile, endocrine profile (including serum ovarian function), electrocardiogram, breast colour Doppler ultrasound and gynaecological colour Doppler ultrasound examinations were conducted at the initial visit and the final follow‐up visit after 3 months. The size of the lump was evaluated by ultrasound examination. Monthly follow‐up visits were conducted to record medication usage, symptom scoring, concurrent medication and adverse events. For details, see Appendices 1 and 2 The clinical trial flowchart (Table A1) and menstrual flow record form (Table A2) are provided in these appendices.

According to the Diagnostic and Therapeutic Efficacy Standards for Traditional Chinese Medicine Diseases [11, 12], a quantitative scoring standard was formulated based on symptom manifestations for comparison of quantitative scores. Breast lump: lump < 1 cm—1 point; 1 cm ≤ lump < 2 cm—2 points; lump ≥ 2 cm—3 points. The hardness score of the lump (measured by touching the breast with the fingertips of the right index finger, middle finger and ring finger): soft texture—1 point; texture as tough as the nose tip—2 points; texture as hard as the forehead—3 points. Breast tenderness: dull pain without tenderness—1 point; hidden pain with tenderness—2 points; pain and tenderness are both significant—3 points; severe pain, unable to touch or affect movement—4 points. Chest tightness: mild chest tightness—0.5 points; chest tightness accompanied by low mood—1 point; chest tightness accompanied by low mood and bloating—1.5 points; no blood clots during menstruation—0 points; a small amount of blood clot—0.5 points; moderate amount of blood clot—1 point; a large amount of blood clots—1.5 points. The menstrual flow was calculated using the weighing method (the weight gain of sanitary pads after use [g] is approximately equal to menstrual flow [mL]). Menstrual flow < 30 mL—menstrual flow is insufficient; 30–80 mL—menstruation is normal; > 80 mL—excessive menstruation.

When laboratory tests indicated abnormal liver and kidney function, coagulation function, endocrine function or abnormal electrocardiogram results, the researcher determined whether the adverse event was caused by the drug. When adverse events occurred, researchers reported them within the prescribed time period and provided necessary medical treatment. Participants who experienced adverse events withdrew from the study.

### 2.3. Treatment Method

The oral administration of Honghua Xiaoyao pills (Jiangxi PROZIN Pharmaceutical Co., Ltd., Jiangxi, China, SFDA approval number: Z20080299) was as follows: the dosage was four pills per dose, three times a day, for a treatment duration of 3 months, without stopping during menstruation. Hormones, analgesics, Western medicine and similar Chinese herbal medicines were not allowed during the trial.

The comprehensive therapeutic efficacy assessment criteria were as follows: (1) clinical cure—the disappearance of breast masses and breast pain, with a main symptom score of 0; (2) significant improvement—a reduction in the main symptom score of ≥ 70% or a reduction in the longest diameter of the mass by > 50%; (3) improvement—a reduction in the main symptom score of ≥ 30% and < 70% or a reduction in the longest diameter of the mass by > 33%; (4) no improvement—not meeting the above criteria. The efficacy assessment criteria for TCM syndromes were as follows: syndrome efficacy rate = ((total score before treatment − total score after treatment)/total score before treatment) × 100%; (1) cure—syndrome efficacy rate after treatment ≥ 90%; (2) significant improvement—syndrome efficacy rate after treatment ≥ 70% and < 90%; (3) improvement—syndrome efficacy rate after treatment ≥ 30% and < 70%; (4) no improvement—syndrome efficacy rate after treatment < 30%.

The efficacy assessment criteria for menstrual volume treatment were as follows: cure—menstrual volume > 30 mL (equivalent to a menstrual bleeding score > 37 points); significant improvement—an increase in menstrual bleeding score of > 70% compared with before treatment; improvement—an increase in menstrual bleeding score of 30%–70% compared with before treatment; no improvement—not meeting the above criteria. The total effective rate was calculated by summing the cure rate, significant improvement rate and effective rate. Baseline data (including symptom scores, TCM syndrome scores and bleeding volume) were collected from participants within 24 h after inclusion in the study. The efficacy evaluation was performed by a specific researcher who was not involved in the patient’s treatment.

### 2.4. Statistical Analysis Methods

Data were analysed using SAS 9.4 software (SAS Institute Inc., Cary City, North Carolina, United States). Descriptive statistics, including mean, standard deviation, median, minimum, maximum, lower quartile, upper quartile and counts and percentages for categorical variables, were calculated for quantitative variables. For comparisons of corresponding variables before and after medication (scores for symptoms such as pain and lumps), paired *t*‐tests (for normally distributed data; normality was assessed using the Kolmogorov–Smirnov test) or Wilcoxon signed‐rank tests were used for quantitative data (efficacy‐ and side effect–related indicators). McNemar’s test or the exact probability method was used for comparisons of categorical data before and after medication, and the Wilcoxon signed‐rank test or the Cochran–Mantel–Haenszel test was used for ordinal data. If > 20% of the data collected for a participant were missing, or if the dosage of therapeutic drugs was < 80%, it was considered as data missing, and the participant was not included in the analysis.

## 3. Results

### 3.1. Treatment Effect

After 3 months of treatment, the average scores of breast pain symptoms, breast lump size symptoms and lump texture symptoms decreased. The scores at 1, 2 and 3 months of treatment were significantly different from the baseline scores (*p* < 0.0001). This indicates a reduction in breast pain symptoms, breast lump size symptoms and lump texture symptoms with treatment. The score significantly decreased after 3 months of treatment compared with 1 month after treatment, which confirms the good long‐term efficacy (Table 1).

**TABLE 1:** Main symptom scores (*n* = 53).

Item	Visit time	Disease score mean ± SD	*p* value (comparison with baseline period)	*p* value (compared to 1 month after intervention)
Breast pain	Baseline	4.21 ± 1.28		< 0.0001
	1 month	2.91 ± 1.3	< 0.0001	
	2 months	1.59 ± 1.17	< 0.0001	
	3 months	0.52 ± 1.08	< 0.0001	< 0.0001

Breast lump	Baseline	1.85 ± 1.05		< 0.0001
	1 month	1.57 ± 0.89	< 0.0001	
	2 months	0.91 ± 0.79	< 0.0001	
	3 months	0.4 ± 0.72	< 0.0001	< 0.0001

Lump texture	Baseline	1.49 ± 0.67		< 0.0001
	1 month	1.15 ± 0.57	< 0.0001	
	2 months	0.64 ± 0.56	< 0.0001	
	3 months	0.33 ± 0.51	< 0.0001	< 0.0001

Total score	Baseline	7.55 ± 2.11		< 0.0001
	1 month	5.62 ± 1.87	< 0.0001	
	2 months	3.13 ± 1.93	< 0.0001	
	3 months	1.25 ± 2.04	< 0.0001	< 0.0001

The scores at 1, 2 and 3 months of treatment were significantly different from the baseline scores, indicating a reduction in secondary symptom scores with treatment. The score significantly decreased after 3 months of treatment compared with 1 month after treatment, which confirms the good long‐term efficacy (Table 2).

**TABLE 2:** Secondary symptom scores (*n* = 53).

Item	Visit time	Disease score mean ± SD	*p* value (comparison with baseline period)	*p* value (comparison with 1 month after intervention)
Lower abdominal pain	Baseline	0.74 ± 0.79		< 0.0001
	1 month	0.45 ± 0.61	< 0.0001	
	2 months	0.17 ± 0.38	< 0.0001	
	3 months	0.15 ± 0.5	< 0.0001	< 0.0001

Breast swelling	Baseline	1.02 ± 0.57		< 0.0001
	1 month	0.36 ± 0.48	< 0.0001	
	2 months	0.09 ± 0.3	< 0.0001	
	3 months	0.09 ± 0.3	< 0.0001	< 0.0001

Blood clots	Baseline	1.3 ± 0.5		< 0.0001
	1 month	1.06 ± 0.57	< 0.0001	
	2 months	0.83 ± 0.64	< 0.0001	
	3 months	0.64 ± 0.63	< 0.0001	< 0.0001

Chest tightness	Baseline	0.28 ± 0.53		< 0.0001
	1 month	0.11 ± 0.38	< 0.0001	
	2 months	0.04 ± 0.19	< 0.0001	
	3 months	0.00 ± 0.00	< 0.0001	< 0.0001

Emotional state	Baseline	1.19 ± 0.62		< 0.0001
	1 month	0.7 ± 0.64	< 0.0001	
	2 months	0.4 ± 0.49	< 0.0001	
	3 months	0.15 ± 0.36	< 0.0001	< 0.0001

In terms of comprehensive therapeutic efficacy, after 3 months of treatment, there were 30 cases (56.6%) of clinical cure, 14 cases (26.4%) of significant improvement, 6 cases (11.4%) of improvement and 3 cases (5.6%) of no improvement. From the perspective of TCM syndromes, after 3 months of treatment, there were 26 cases (49.1%) of cure, 15 cases (28.4%) of significant improvement, 9 cases (16.9%) of improvement and 3 cases (5.6%) of no improvement (Table 3).

**TABLE 3:** Therapeutic efficacy (*n* = 53).

		Number (*N*)	Percentage (%)
Comprehensive therapeutic efficacy	Cure	30	56.6
	Significant improvement	14	26.4
	Improvement	6	11.4
	No improvement	3	5.6

Traditional Chinese medicine efficacy	Cure	26	49.1
	Significant improvement	15	28.4
	Improvement	9	16.9
	No improvement	3	5.6

### 3.2. Side Effects

There were no statistically significant changes in menstrual volume or duration (*p* > 0.05) after intervention (Table 4). The coagulation profile, hormone profile, endometrial thickness and arterial indicators did not show statistically significant changes (*p* > 0.05) after the intervention (Table 5). No adverse events or reactions were observed during the study. Safety indicators in laboratory tests did not show clinically significant changes.

**TABLE 4:** Menstrual status (*n* = 53).

Item	Visit time	Disease score mean ± SD	*p* value
Menstrual volume (mL)	Baseline	106.09 ± 71.71	
	1 month	97.51 ± 56.33	0.88657
	2 months	108.21 ± 64.16	0.25728
	3 months	100.17 ± 64.85	0.92134

Menstrual duration (days)	Baseline	5.7 ± 1.22	
	1 month	5.64 ± 1.08	0.52051
	2 months	5.6 ± 1.12	0.44069
	3 months	5.83 ± 1.22	0.60144

**TABLE 5:** Changes in coagulation indices before and after treatment.

		Baseline	After 3 months	Difference	*p* value
Coagulation function	Prothrombin time	11.51 ± 0.69	11.66 ± 0.43	0.15 ± 0.66	0.10799
	Partial thromboplastin time	28.26 ± 2.19	28.61 ± 2.26	0.35 ± 2.18	0.24608
	Fibrinogen	2.46 ± 0.47	2.5 ± 0.5	0.04 ± 0.31	0.34965
	Thrombin time	16.98 ± 1.5	17.25 ± 1.35	0.27 ± 2.06	0.35364

Hormones	Prolactin	412.95 ± 229.55	419.7 ± 243.85	6.75 ± 295.35	0.86855
	Follicle‐stimulating hormone	19.49 ± 92.77	6.8 ± 2.28	−12.69 ± 92.68	0.32346
	Luteinizing hormone	5.57 ± 2.11	8.25 ± 12.15	2.67 ± 12.38	0.12218
	Estradiol	173.5 ± 224.29	209.25 ± 297.97	35.75 ± 360.43	0.47344
	Progesterone	0.94 ± 0.5	1.87 ± 5.7	0.94 ± 5.74	0.24135
	Anti‐mullerian hormone	3.73 ± 1.85	3.81 ± 2.04	0.08 ± 1.16	0.63691

Uterine b‐ultrasound	Endometrial thickness	0.83 ± 0.24	0.86 ± 0.24	0.03 ± 0.23	0.39896
	Uterine artery resistance index (Left)	0.83 ± 0.07	0.82 ± 0.06	−0.01 ± 0.09	0.38501
	Uterine artery pulsatility index (Left)	2.35 ± 0.62	2.34 ± 0.62	0 ± 0.74	0.99243
	Uterine artery pulsatility index (Right)	2.38 ± 0.71	2.31 ± 0.53	−0.07 ± 0.78	0.5176
	Right gland thickness	1.37 ± 0.43	1.3 ± 0.41	−0.07 ± 0.3	0.10937
	Left gland thickness	1.34 ± 0.44	1.31 ± 0.42	−0.03 ± 0.28	0.44407

Complete blood count	Haemoglobin	129.86 ± 8.86	131.28 ± 10.6	1.81 ± 7.43	0.08176
	White blood cell count	5.09 ± 1.05	5.29 ± 1.34	0.23 ± 1.17	0.15486
	Lymphocyte count	1.74 ± 0.45	1.74 ± 0.51	0 ± 0.38	0.97132
	Neutrophil count	2.92 ± 0.85	11.07 ± 57.54	8.19 ± 57.46	0.30447
	Platelet count	265.11 ± 60.95	255.43 ± 70.53	−9.53 ± 68.24	0.31384

Liver function	Alanine aminotransferase	11.9 ± 6.29	12.68 ± 7.49	0.9 ± 6.14	0.29047
	Aspartate aminotransferase	15.38 ± 3.21	17.81 ± 15.93	2.35 ± 16	0.29039
	Total bilirubin	9.25 ± 4.99	9.8 ± 4.43	0.57 ± 3.54	0.24637
	Total serum protein	74.54 ± 3.91	73.56 ± 4.56	−0.92 ± 4.81	0.16953

Kidney function	Creatinine	58.86 ± 6.65	59.45 ± 8.79	0.54 ± 7.66	0.6104
	Uric acid	263.95 ± 61.28	262.97 ± 65.93	0.14 ± 50.37	0.98426

## 4. Discussion

The incidence of HMG is fairly high, with an estimated 50%–70% of women experiencing HMG at some point in their lives [13]. The incidence of HMG increases with age, especially in women aged 30–50 years [14]. HMG has wide‐ranging effects on patients, including physiological, psychological, quality of life, cancer risk and social impacts [15]. Timely treatment of HMG to improve symptoms and enhance quality of life is of great significance. The pathogenesis of HMG is closely associated with factors such as endocrine disorders, emotional fluctuations and unhealthy lifestyle habits [16].

Liver depression and haemostasis type is a common subtype of HMG, characterised by emotional depression, irritability, chest tightness, pain in the sides of the body and menstrual irregularities [17]. TCM has great efficacy in the treatment of liver depression and haemostasis‐type HMG. Some TCM preparations, such as breast‐reducing granules, have better safety than the Western medicine tamoxifen and have better therapeutic effects in relieving pain, eliminating lumps and other aspects [18]. Honghua Xiaoyao Pian was developed based on the ancient Chinese formula ‘Xiaoyao San’, with the omission of ginger and the addition of safflower and Zaojiaoci. Its main ingredients include Chinese angelica, white peony root, white atractylodes rhizome, *Poria cocos*, safflower, Chinese honeylocust spine, *Bupleurum marginatum*, mint and liquorice. It is used for treating symptoms such as discomfort in the liver, distending pain in the chest and sides, dizziness, decreased appetite and menstrual irregularities [9]. Safflower helps to improve blood circulation in the breast and reduce inflammatory reactions. Chinese angelica can regulate menstruation and relieve pain, thereby helping to adjust hormone levels and alleviate the impact of hyperplastic mammary glands on the patient’s quality of life [19–21]. Red peony root can help alleviate symptoms such as breast tenderness and lumps [22, 23]. *Bupleurum marginatum* can help alleviate symptoms such as emotional depression and irritability [24, 25]. *Poria cocos* can improve water metabolism and reduce oedema. Liquorice has the function of reducing drug toxicity and improving the efficacy of the medication [26, 27]. These Chinese herbal ingredients interact with each other, achieving the purpose of treating liver depression and haemostasis of HMG.

In recent years, many studies have examined Honghua Xiaoyao pills in the treatment of gynaecological diseases. These studies mainly evaluate the therapeutic effects of Honghua Xiaoyao pills by observing patients’ clinical symptoms, signs and imaging examination results, such as breast ultrasound and mammography [28, 29]. This study found that Honghua Xiaoyao pills can alleviate breast pain, reduce breast lump size and improve lump texture. They also significantly improve symptoms such as lower abdominal pain, menstrual blood clots, emotional instability and chest tightness. Long‐term (3 months) medication has better results. This study showed that the reasonable use of Honghua Xiaoyao pills during menstruation did not have a significant impact on menstrual volume, duration, hormone levels or coagulation function. The mechanism of the Honghua Xiaoyao pill’s treatment of HMG was to regulate hormone disorders. Honghua Xiaoyao pills could reduce oestrogen‐stimulated hyperplasia by inhibiting the production of its precursor androgen [30].

However, there were some limitations in this study. First, there was no comfort group or standard treatment group set up, which limited the ability to attribute observed improvements solely to the intervention. Second, a small sample size limited statistical power and generalisability, and the 3‐month treatment period may not be long enough to assess the long‐term efficacy and safety of the intervention fully. Third, a lack of blinding and objective measurements may have resulted in biased results. In future research, we recommend a larger, multicentre, randomised controlled trial and mechanistic studies to understand how Honghua Xiaoyao pills work.

Honghua Xiaoyao pills have potential value in the treatment of liver depression and haemostasis‐type HMG; however, further research is needed to clarify their mechanism of action and optimise treatment strategies, thereby providing better support for clinical practice.

## Author Contributions

Concept: Xiao‐Jun Zhang.

Data management: Li Zheng and Wei Liu.

Data analysis: Jia‐Ying Qiu, Yang Zhao, Xiao‐Feng Li, Zhi‐Gang Zhou, Jiang‐Tao Wang and Yu Cui.

Funding acquisition: Xiao‐Jun Zhang.

Research implementation: Xiao‐Feng Li and Zhi‐Gang Zhou.

Method development: Li Zheng, Wei Liu and Xiao‐Jun Zhang.

Project management: Xiao‐Feng Li and Xiao‐Jun Zhang.

Resource provision: Zhi‐Gang Zhou.

Software development: Li Zheng.

Supervision: Wei Liu.

Verification: Xiao‐Feng Li.

Content preparation: Li Zheng and Wei Liu.

Initial draft writing: Li Zheng and Wei Liu.

Modification and Comment: Li Zheng, Wei Liu, Jia‐Ying Qiu, Yang Zhao, Xiao‐Feng Li, Zhi‐Gang Zhou, Jiang‐Tao Wang, Yu Cui and Xiao‐Jun Zhang.

## Funding

This study did not receive any funding in any form.

## Ethics Statement

This study was conducted in accordance with the Declaration of Helsinki and approved by the Ethics Committee of Xiyuan Hospital of China Academy of Chinese Medical Sciences (Approval No: 2021XLA077‐2).

## Consent

The authors have nothing to report.

## Conflicts of Interest

Researchers affiliated with drug manufacturers only participate in adverse reaction monitoring, jointly determine adverse events with attending physicians, and are responsible for reporting adverse events. They do not participate in other processes of the research. The other authors declare no conflicts of interest.

## Data Availability

All data generated or analysed during this study are included in this article. Further enquiries can be directed to the corresponding author.

## Appendix 1

.

**Table A1:** Clinical trial flowchart.

Item study phase	Visit 1 (baseline)	Visit 2 (1 month)	Visit 3 (2 months)	Visit 4 (3 months after menstrual cycle, days 5–7)
Informed consent signed	√			
Medical history, demographic data	√			
TCM syndrome	√	√	√	√
General physical examination	√	√	√	√
Record menstrual status	√	√	√	√
Complete blood count	√			√
Urinalysis	√			√
Liver and kidney function	√			√
Coagulation profile	√			√
Endocrine profile + serum ovarian function	√			√
Electrocardiogram	√			√
Urinary pregnancy test	√			√
Breast ultrasound	√			√
Gynaecological ultrasound	√			√
Confirm inclusion and exclusion criteria	√			
Dispense investigational product	√	√	√	
Drug collection		√	√	√
Adverse event record		√	√	√
Concomitant medication record	√	√	√	√

*Note:* 1. Urine pregnancy test is limited to women of reproductive age between 18 and 50 years old. 2. Complete blood count: White blood cells, neutrophils, lymphocytes, red blood cells, haematocrit, haemoglobin, platelets. 3. Urinalysis: Urine protein, urine occult blood, urinary red blood cells, urinary white blood cells, urine specific gravity. 4. Liver and kidney function: Alanine aminotransferase, aspartate aminotransferase, total bilirubin, blood creatinine, uric acid, blood urea nitrogen. 5. Coagulation function: Including fibrinogen, prothrombin time, thrombin time, and partial thromboplastin time. 6. Endocrine profile should be measured on an empty stomach between days 2–4 of the menstrual cycle; gynaecological ultrasound should be performed after the completion of the menstrual period.

## Appendix 2

.

**Table A2:** Menstrual flow record form.

Day sanitary pad				Large blood clots	Small blood clots	Blood clots lost
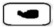
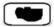
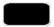
Day 1						
Day 2						
Day 3						
Day 4						
Day 5						
Day 6						
Day 7						

*Note:* 1. The scoring criteria for menstrual blood loss are as follows: ①Bleeding area infiltrating ≤ 1/3 of the entire sanitary pad surface: 1 point per pad; ②Bleeding area infiltrating 1/3 to 3/5 of the entire sanitary pad surface: 5 points per pad; ③Bleeding area essentially covering the entire sanitary pad: 20 points per pad; ④Blood clots smaller than the size of a 1‐yuan coin: 1 point per clot; ⑤Blood clots larger than the size of a 1‐yuan coin: 5 points per clot; ⑥Estimate the amount of menstrual blood lost during bathing or toileting and record it as a fraction of the total recorded volume for the day. 2. It is recommended to change the sanitary pad when the area of menstrual blood infiltration on the pad accounts for 30%–90% of the leakage prevention ring area or when it has been used for more than 3 h. 3. Use designated “Sofy” brand sanitary pads during menstruation, with a length of 23 cm (for sleeping, two pads of the specified size can be used together to prevent leakage).
